# Fractal feature selection model for enhancing high-dimensional biological problems

**DOI:** 10.1186/s12859-023-05619-z

**Published:** 2024-01-09

**Authors:** Ali Hakem Alsaeedi, Haider Hameed R. Al-Mahmood, Zainab Fahad Alnaseri, Mohammad R. Aziz, Dhiah Al-Shammary, Ayman Ibaida, Khandakar Ahmed

**Affiliations:** 1 Present Address: College of Computer Science and Information Technology, University of Al-Qadisiyah, Diwaniyah, 58009 Iraq; 2 Department of Computer Techniques, Imam Kadhum College, Diwaniyah, 58009 Iraq; 3 Department of Computer Science, College of Science, University of Mustansiriyah, Baghdad, 10052 Iraq; 4 Intelligent Technology Innovation Lab, Victoria University, Melbourne, VIC Australia

**Keywords:** Bioinformatics, Feature selection, High-dimensional datasets, Fractal, Machine learning

## Abstract

The integration of biology, computer science, and statistics has given rise to the interdisciplinary field of bioinformatics, which aims to decode biological intricacies. It produces extensive and diverse features, presenting an enormous challenge in classifying bioinformatic problems. Therefore, an intelligent bioinformatics classification system must select the most relevant features to enhance machine learning performance. This paper proposes a feature selection model based on the fractal concept to improve the performance of intelligent systems in classifying high-dimensional biological problems. The proposed fractal feature selection (FFS) model divides features into blocks, measures the similarity between blocks using root mean square error (RMSE), and determines the importance of features based on low RMSE. The proposed FFS is tested and evaluated over ten high-dimensional bioinformatics datasets. The experiment results showed that the model significantly improved machine learning accuracy. The average accuracy rate was 79% with full features in machine learning algorithms, while FFS delivered promising results with an accuracy rate of 94%.

## Introduction

**Fig. 1:**
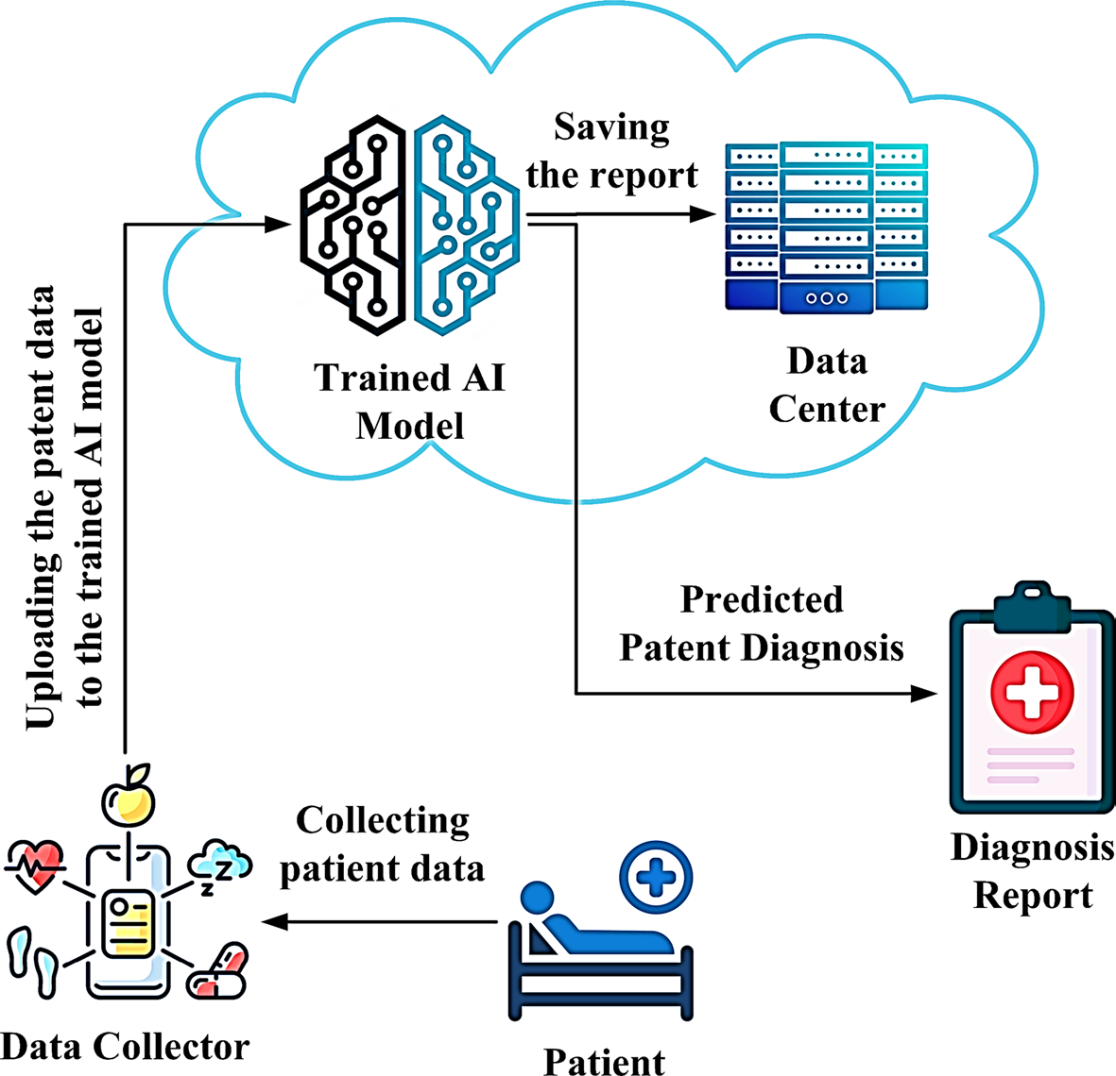
Proposed scenario of intelligent model for diagnosis of patient state

Bioinformatics is an interdisciplinary field that combines biology, computer science, and statistics to analyze and interpret biological behaviour [1]. It identifies and diagnoses cancer by examining gene activity and cellular function. Gene expression profiling (GEP) is a helpful description used in bioinformatics to measure the activity of thousands of genes simultaneously, providing a comprehensive picture of cellular function in a particular biological sample [2]. However, this wealth of molecular information presents a unique challenge and opportunity for the field of artificial intelligence [3]. The confluence of big data and high-dimensional datasets poses a daunting challenge to the machine-learning community, highlighting the complexity of performance versus feature reduction or selection. When faced with unprocessed Big Data and high-dimensional datasets without feature reduction or section, the performance of machine learning algorithms shows complicated implications [4]. The lack of a readout mechanism increases computational overhead as algorithms struggle with unwieldy data representations, which impacts efficiency [5, 6]. In addition, the unrefined data environment leads to increased susceptibility to overfitting, where models overfit the peculiarities of the training data, compromising their ability to generalize to unknown instances [7–10]. Without proper feature reduction or selection, models struggle with redundant, irrelevant, or noisy features, reducing their ability to find meaningful patterns in the data [11]. Figure 1 illustrates the proposed Scenario of applied AI for predicting the diagnosis based on analysis of the bioinformatics of the patient.

Two different feature selection techniques, the filter and wrapper models, are a hallmark of improving model efficiency and performance. With their respective strengths, these methods provide complementary approaches for selecting relevant attributes.

The domain of feature selection extensively employs statistical methodologies to distil relevant attributes from complex datasets [12]. These techniques leverage correlation, significance, and variability measures to uncover the pivotal dimensions that drive model performance. Using statistical models for feature selection has several advantages. These models can help identify the most essential features in a dataset, reduce the dimensionality of the data, and improve the performance of machine learning algorithms [13–15]. However, wrapper models for feature selection can have drawbacks, such as randomness and unstable results. Wrapper models rely on a specific learning algorithm to evaluate the importance of features, which can lead to biased results and overfitting [16]. The concept of fractals provides a mathematical framework for describing and revealing the relationships among patterns in the data [17]. A fractal is a geometric shape or design characterized by its complex self-similarity across different scales [18]. This unique property of fractals underpins the understanding of complex, self-replicating structures and offers insights into their behaviour in various scientific contexts. Building on this fundamental understanding, integrating the fractal concept into data analysis unlocks the potential to decipher intricate patterns and structures in complex datasets [19]. The strength of this approach lies in its ability to capture self-similarity and hierarchical relationships across different scales, allowing hidden relationships to be detected even in high-dimensional data. Considering the complexity of data in high-dimensional problems and the importance of selecting meaningful features, the FFS method is the best choice. Given the complexity of data in high-dimensional issues and the critical importance of choosing significant features, this paper introduces fractal feature selection (FFS). This innovative method is inspired by fractal behaviour and explicitly targets the challenges conventional feature selection methods face. When examining features of the dataset, a high correlation coefficient indicates a strong relationship between features and the target in the dataset. By conceptualizing these attributes as blocks, where each block corresponds to a particular data category, the proposed model finds that blocks with common similarities are often associated with specific data categories. The true power of the proposed FFS lies in its ability to mitigate traditional models' inherent randomness and unpredictability. Rather than being constrained by a limited search parameter, FFS penetrates deeper into the data set. It broadens its analytical horizons and identifies hidden relationships and nuances with precision.

### Motivation

Feature selection enables data modelling efficiency by eliminating redundant inputs, leading to faster execution and enhanced model performance.[20]. Feature selection uses a variety of models, including statistically based approaches, wrapper methods, and intrinsic methods. Feature selection refines the analysis process and leads to more efficient, accurate, and interpretable results. The main goal of modern feature selection models is to enhance system performance by strategically removing redundant attributes, thereby streamlining the analysis process.[21]. The current feature selection model, or wrapper or filter, has several limitations that can be summarized as follows.

1. *Instability*: While promising, these feature selection models grapple with intrinsic limitations that impact their efficacy. One notable concern pertains to the randomness introduced by the instability of system performance. This randomness introduces an element of unpredictability, potentially undermining the reliability of the feature selection process [22].
2. *Constrained search space*: Another limitation arises from the limited search space in which these models operate. The search space, usually between 0 and 1, can lead to a stagnation scenario that hinders the comprehensive exploration of optimal feature subsets [23].
3. *Integration of metaheuristics*: A promising way to overcome these limitations is to integrate metaheuristics into different aspects of the system [2, 24]. The strategic application of metaheuristics has led to tangible improvements that address the challenges of unstable performance and expand the search space for more robust exploration.

### Contribution

The proposed fractal feature selection (FFS) model revolutionizes data analysis, offering a streamlined system for high-performance, accurate, and stable feature selection. The contributions in the proposed FFS are summarized as follows:

1. *Accurate and stable feature selection*: The proposed FFS model can perform feature selection with high accuracy and stability using fractal concepts. It selects highly relevant features that improve predictive ability while reducing the risks associated with noisy or irrelevant features. Moreover, the proposed FFS model is stable regarding sets of features and the performance of outcome results.
2. *Efficient prediction through low complexity*: The proposed fractal feature selection (FFS) model is proof of harmonic convergence of a low-complexity system with remarkable performance. Through the sophisticated integration of fractal analysis, the FFS model can deftly navigate the intricacies of high-dimensional data while maintaining computational efficiency. The model achieves deep understanding without succumbing to computational overhead by detecting underlying self-similarities and hierarchies within the data. This balance between simplicity and predictive accuracy makes the FFS model an innovative solution that redefines the data analytics landscape through seamless integration.
3. *High-relevant features*: The proposed model is unique in achieving efficient prediction by selecting highly correlated features. The model can improve its predictive ability by identifying and selecting the most relevant features while reducing the risks associated with noisy or irrelevant features.

### Evaluation strategies

Evaluation strategies use the analysis of confusion matrices and the extraction of essential parameters to evaluate the accuracy and usefulness of feature selection models. Precision, recall, F1 score, and specificity provide a detailed assessment of model performance. The correlation coefficient metrics are used to test the validity of the features selected by the proposed FFS. Furthermore, comparing the proposed FFS model with current models highlights its uniqueness and confirms its potential to advance the field of feature selection.

### Paper organization

The paper is divided into several sections. Section “Related works” discusses related work in the field. Section “Feature selection” deals with the selection of features for the proposed model. Section “Problem formulation” defines the problem to be solved by the model, and Sect. “Proposed technique” presents the model in detail. Section “Experiments and discussion” presents and discusses the results of the experiments conducted to evaluate the model. Finally, Sect. “Discussion” concludes the paper and suggests possible areas for future research.

## Related works

This section analyses previous research addressing feature selection, which is an essential component of data modelling and aims to reduce the number of input variables to enhance the model's efficiency and effectiveness. Various feature selection approaches have been proposed, including statistically based wrappers and intrinsic methods. Each process has advantages and disadvantages, and ongoing research focuses on developing more accurate and robust feature selection models.

In Wei et al. [12], a feature selection model was proposed based on the maximum mutual information and entropy of features to select appropriate features. The proposed model uses a hybrid method based on dynamic feature importance, which evaluates the relevance of each feature in the context of data analysis, thereby improving the accuracy and effectiveness of feature selection in the given research framework. However, a limitation of this method is that low redundancy is not a crisp parameter for deciding on features with high significance values.

Parvasideh et al. [25] used a dictionary-learning algorithm for feature selection. It uses a total least squares approach to rank and select features. The authors set the parameter (k) as the number of features to be selected when the k-features have minimum parameters. While it holds the potential to enhance feature selection accuracy, it comes at the cost of heightened computational complexity. This aspect warrants careful consideration, particularly in contexts demanding efficient analyses, as the model's intricate computations may hinder real-time applicability. So, the research shows how hard it is to find a good balance between speed and accuracy when making feature selection strategies for high-dimensional datasets.

In the study in Adorada et al. [26], the authors used Support Vector Machine-Recursive Feature Elimination (SVM-RFE) for feature selection. This approach uses Support Vector Machines (SVMs) to eliminate less relevant features iteratively. The proposed model removed features that contribute less to the discrimination process based on recursive feature elimination (RFE), and SVM is an objective function of the proposed SVM-RFE model. The inherent randomness associated with the SVM-RFE process introduces system performance instability. This instability could potentially affect the consistency and reliability of the feature selection results.

Al-Shammary et al. [2] introduced the extended particle swarm optimization (EPSO) model, potentially improving the PSO search process for optimization problems. The model is applied to gene expression profiles, important molecular biology measurement factors used in cancer diagnosis. A modified wrapper feature selection model is applied to address the gene classification challenge by replacing the random approach with EPSO. However, the reliance on controlled randomness could introduce a level of complexity that could reduce the reproducibility and reliability of the results of the proposed model.

In Ibrahim et al. [27], the Harris-Hawks optimizer was modified for feature selection and the support vector machine as an object function. The authors propose a hybrid strategy based on the Harris-Hawk optimization (HHO) algorithm to optimize the parameters of the SVM model and find the optimal feature subset. The proposed model relies on random levels for operations, and this approach increases instability and unpredictability. Therefore, the proposed model's complexity and potential reduced the results' reliability.

Gao et al. [28] and [29] address the issue of feature redundancy in information-theoretical-based feature selection methods, where larger values of the traditional feature redundancy term do not necessarily indicate worse candidate features. The authors propose a new feature redundancy term that considers the relevancy between a candidate feature and the class given each already-selected feature called min-redundancy and max-dependency (MRMD). The proposed model relies on multiple algorithms for feature selection and classification. Additionally, it employs various machine learning algorithms for both tasks, which adds complexity to the system.

In their paper, Zhang et al. [30] introduced a feature selection approach known as the Maximal Independent Classification Information and Minimal Redundancy (MICIMR) algorithm. The algorithm determines the relevance and redundancy terms of class-independent features using the symmetric uncertainty coefficient and the relevance and redundancy terms of class-dependent features based on the independent classification information criterion. However, there are limitations to this model. Selecting features with high classification information may result in redundancy where multiple features provide similar or overlapping data. On the other hand, reducing redundancy may result in leaving out individually powerful features in classification.

Wang et al. [31] introduced a method for dimensionality reduction that combines feature selection and feature extraction using fuzzy rough set theory. The Feature Set Partition-based approach to Fuzzy Rough Dimensionality Reduction (FSPFRdr) aims to fully consider the intrinsic information contained in features and differentiate the significance level between them. The original feature set is divided into three categories: nonsignificant, weakly significant, and significant features, based on the normalized independent classification information (NICI). The nonsignificant features are removed before dimensionality reduction. In contrast, the weakly important features are processed using the proposed Fuzzy Similarity Relation-based Supervised Locally Linear Embedding (FRSLLE) to obtain an embedded feature set. However, the proposed fuzzy rough model is unsuitable for dynamic and multi-label data, negatively impacting its effectiveness.

Thakkar et al. [32] present an approach that integrates statistical significance to enhance feature selection in Deep Neural Networks (DNNs) for Intrusion Detection Systems (IDS). This method aims to optimize the performance of DNN-based IDS by selecting only the most relevant features. The limitation of this work is that the authors employ deep learning as their objective function, inadvertently increasing the model's time complexity. Moreover, deep learning models typically require substantial features to function optimally, which could counteract the benefits of feature reduction.

The authors in [33] present SemiACO combine semi-supervised learning with ant colony optimization for feature selection. The model demonstrates the potential of using nature-inspired algorithms in feature selection, but a limitation arises from the inherent complexity of ant colony optimization. This can increase computational costs, especially when managing large and complicated datasets. Furthermore, while ant colony optimization is adept at finding solutions, it does not always guarantee convergence to the global optimum, depending on the problem landscape and the algorithm's parameters.

Table 1 summarizes the related works regarding datasets, proposed models, and achieved accuracy.

**Table 1:** Summarizes the related works

Refs	Name of proposed models	Datasets	Accuracy
[2]	hybrid feature selection method Dynamic Feature Importance (DFI)	Biological data Face image data Biological data Other data	85.01 ± 0.12 98.33 ± 0.54 98.86 ± 0.87 87.32 ± 0.80
[12]	A robust dictionary learning based on total least squares (ITLS-Robust)	SMK-CAN-187 TOX-171 GLI-85 CLL-SUB-111	65.8 65.6 87.5 62.3
[25]	Support vector machine-recursive feature elimination (SVM-RFE)	N/A	N/A
[26]	Extended particle swarm optimization	Biomedical data	100
[27]	Modified Harris Hawks optimizer for feature selection	Real biomedical datasets	100
[28]	a hybrid feature selection method named Minimal Redundancy-Maximal New Classification Information (MR-MNCI)	Biomedical data	94.89
[29]	min-redundancy and max-dependency (MRMD)	N/A	N/A
[30]	Maximal independent classification information and minimal redundancy (MICIMR)	Biomedical data	100
[31]	FSPFRdr and FRSLLE	Biomedical data	95.88 ± 0.41
[32]	fusion of statistical importance using Standard Deviation and Difference of Mean and Median	NSL-KDD, UNSW_NB-15, CIC-IDS-2017	99.84 89.03 99.80
[33]	A semi-supervised feature selection based on ant colony optimization	Biomedical data	N/A

## Feature selection

Feature selection is technically an essential step in data modelling that involves reducing the number of input variables to improve efficiency and effectiveness [21]. It acts as a strategic filter, sifting through the available features and filtering out those that contain the most relevant and meaningful information. This process improves the computational efficiency of the model and contributes to its interpretability and generalizability [9]. It is imperative when dealing with datasets with many variables, where irrelevant or redundant features may introduce noise and complexity, affecting the model's performance. Several techniques are used for feature selection, which are classified based on the functionality of the wrapper and filter models. Filter methods work without relying on predictive models. These methods speed up the feature selection process and are particularly beneficial when faced with high-dimensional datasets [12].

**Fig. 2:**
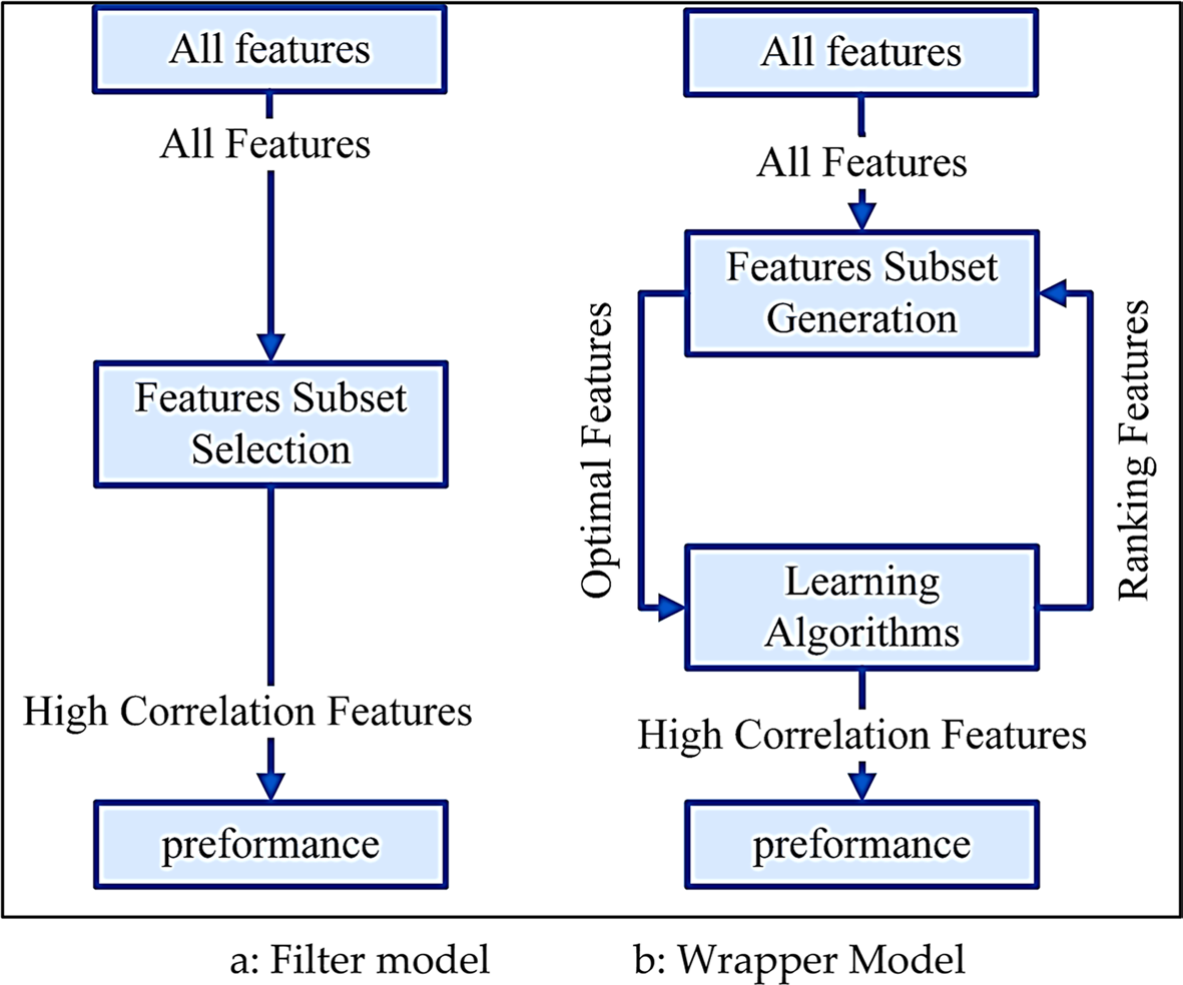
A Comparative Framework of Filter and Wrapper model

In contrast, wrapper methods take a more dynamic approach, assessing the utility of features based on their performance in the context of a particular classifier. In these methods, the feature selection process is essentially (wrapped) around the model itself, iteratively training and evaluating the model as various subsets of features are examined [2]. This approach often produces better results because the model's predictive power is used as the guiding criterion [15, 34, 35]. However, this comes at the cost of increased computational complexity, as the underlying model must be trained and evaluated at each iteration. Figure 2 shows summarizing the comparative essence of these techniques and shows their synergistic interplay in the feature selection process.

## Problem formulation

Feature selection can be conceptualized mathematically, where the dataset comprises several features (x), each represented by a vector of instances (i). This can be expressed as:

1 $$X = \left\{ {x_{1} ,x_{2} ,x_{3} , \ldots \ldots ,x_{n} } \right\}$$

Each instance class can be denoted as in Eq. (2)

2 $$C_{i} = x_{i,1} \cup x_{i,2} \cup x_{i,3} \ldots \ldots \ldots \cup x_{i,n}$$

where n is the number of features, the mathematical representation of the objective function in feature selection is shown in Eq. (3).

3 $$Objective \, Function = f\left( Selected \, Features \, of \, \left( X \right) \right)$$

where $f$ embodies a function mapping selected features to a scalar value signifying the efficacy of the chosen subset. The definition of $f$ pivots on the specific goals and evaluation metrics of the feature selection challenge, such as accuracy, precision, recall, F1-score, mutual information, etc.

## Proposed technique

**Fig. 3:**
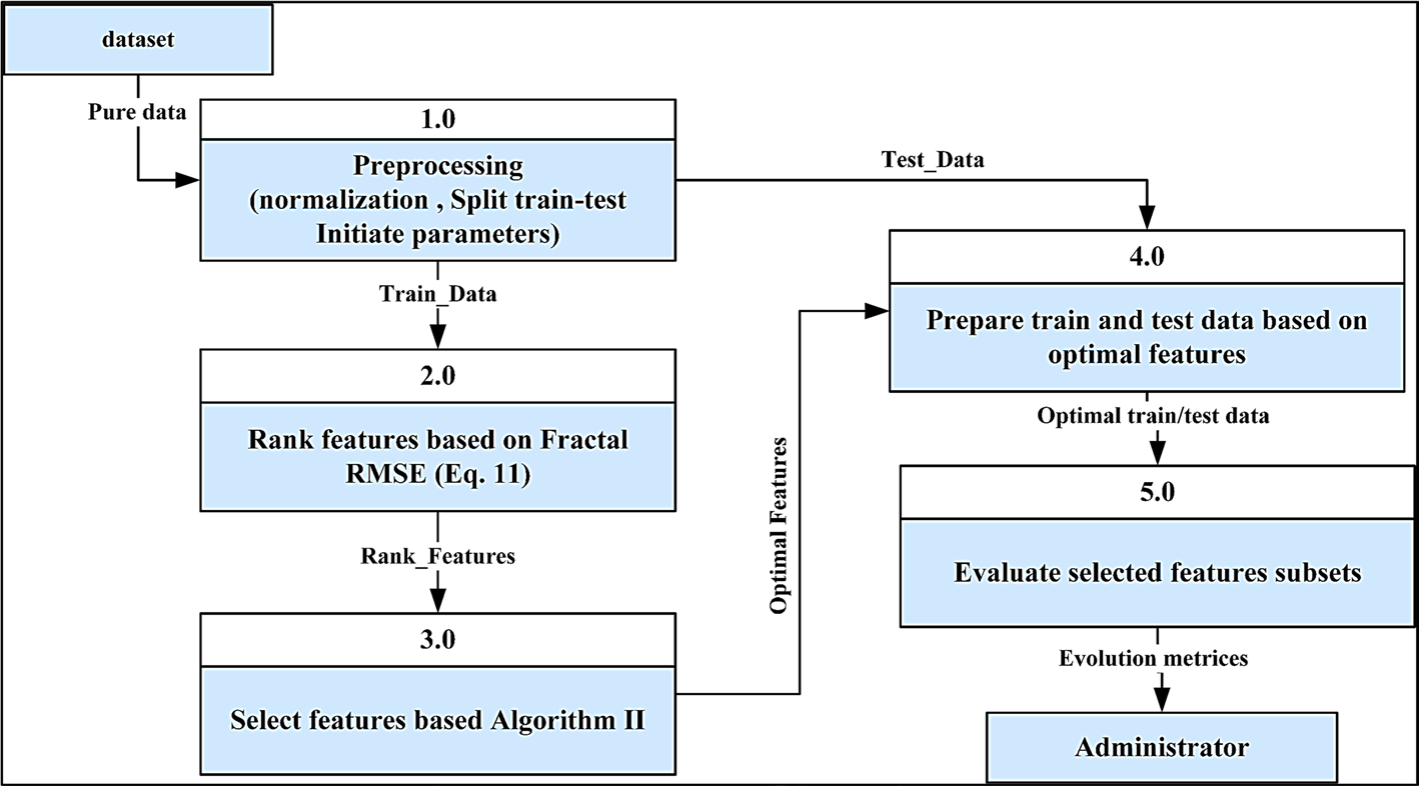
Main steps of the proposed fractal feature selection (FFS) model

Figure 3 depicts the essential constituents of the fractal feature selection (FFS) model designed to optimize high-dimensional biological challenges. The initial three elements of the model are dedicated to preprocessing bioinformatics data, culminating in forming a numeric dataset. Subsequently, the succeeding three components revolve around the incorporation of fractal functions. This framework of the proposed FFS starts with the computation of fractal coefficients and culminates in the judicious selection of features from the numeric dataset objects. The basis for this selection is based on the histograms of fractal root mean square error metric (RMSE).

### Preprocessing data

The first step of the proposed FFS is data preprocessing, which includes several essential tasks. These tasks involve normalizing the data, dividing it into training and test groups, and initializing the parameters. Normalization is a crucial part of this process, and the min–max normalization method is used. It scales the data to a specific range, usually between [0 and 1], to ensure that all features in the model have the same importance. Equation (4) illustrates the normalization phase of the proposed model.

4 $$x_{i}{\prime} = \frac{{x_{i} - min\left( X \right)}}{max\left( X \right) - min\left( X \right)}$$

where $x$ represents the original set of data points within the feature, with $min(X)$, $max\left(X\right)$ denoting the minimum and maximum values within that set, respectively. After normalization, the data is split into training and testing sets, and the model's parameters are initialized to prepare for the next steps in the modelling process.

### Rank features based on fractal RMSE

A fractal is a mathematical object that exhibits self-similarity at different scales [19]. In other words, the structure of the fractal appears similar when viewed at different magnification levels. The parameters of a fractal include range (R), domain (D), and offset (O). Equation (5) shows the relationship among fractal parameters:

5 $$R = S \times D + O$$

The feature selection of the proposed model is based on finding features with high similarity measures. Technically, a fractal represents the instances of the features as spaces, and the model has an objective function. A non-zero value means that the feature has high similarity in its mapping. A low similarity of the feature instances logically implies that the data is scattered in the feature, which does not contribute to the representation of the individual classes. The features that have low similarity have low RMSE. Technically, the essence of RMSE in fractals emanates from analyzing the relationship between the range and the domain. Within the structure of the fractal, the ($D$) is fashioned from the ($R$) to constitute a smaller set that mirrors the characteristics of the ($R$). Therefore, if the intention is to configure the ($R$) from the ($D$), it becomes imperative to interplay it with the scale and the ($O$). According to [36], the formulation of fractal parameters scale, domain, offset, and RMSE are used in the proposed FFS. Equation (6) elucidates the process of sculpting the domain ($D$) from the data within the realm of feature selection for the proposed system.

6 $$\mathop{D}\limits^{\rightharpoonup} _{i} = \mathop {\bigcup }\limits_{j = 0}^{K - 1} \left( {\frac{{\mathop \sum \nolimits_{{m_{1} }}^{{m_{2} }} x_{i} }}{b}} \right)$$

The size ($D$) is found by calculating the $K$ in Eq. (8) and interval bounders [${m}_{1}, {m}_{2}$] of ($D$) in Eqs. (9) and (10), respectively

7 $$K = int\left( \frac{n}{b} \right) + Mod\left( {b, n } \right)$$

where $n$ refers to the number of features, $b$ is a block size

8 $$m_{1} = j \times b + 1$$

9 $$m_{2} = m_{1} + b$$

where $j$ refers to the index of the block. The scale ($S$) parameter is crucial in exploring fractals, allowing for greater magnification and a better understanding of their complex structure. It determines the level of detail visible at any given magnification level, enabling users to zoom in or out to view intricate details or the overall shape of the fractal. Fractal explorers can navigate and comprehend these fascinating mathematical objects by controlling this parameter. Equation (11) calculates the ($S$) of the corresponding feature.

10 $$S = \frac{{n\mathop \sum \nolimits_{i = 1}^{n} d\left( {p_{i} } \right)r\left( {p_{i} } \right) - \mathop \sum \nolimits_{i = 1}^{n} d\left( {p_{i} } \right)\mathop \sum \nolimits_{i = 1}^{n} r\left( {p_{i} } \right)}}{{n\mathop \sum \nolimits_{i = 1}^{n} d\left( {p_{i} } \right)^{2} - \left( {\mathop \sum \nolimits_{i = 1}^{n} d\left( {p_{i} } \right)} \right)^{2} }}$$

where d(pi) represents the value of the *i*th item within the numeric entity *D*, and r(pi) signifies the value within R.

The offset parameter ($O$) that is used to format the ($R$) in the fractal concept is calculated in Eq. (12).

11 $$O = \frac{1}{n}\left( {\mathop \sum \limits_{i = 1}^{n} r\left( {p_{i} } \right) - S\mathop \sum \limits_{i = 1}^{n} d\left( {p_{i} } \right)} \right)$$

The determination of all fractal parameters necessitates the calculation of RMSE through the utilization of Eq. (13).

12 $$RMSE = \sqrt {\frac{1}{n}\left[ {\mathop \sum \limits_{i = 1}^{n} r\left( {p_{i} } \right)^{2} + S\left( {S\mathop \sum \limits_{i = 1}^{n} d\left( {p_{i} } \right)^{2} - 2\mathop \sum \limits_{i = 1}^{n} d\left( {p_{i} } \right)r\left( {p_{i} } \right) + 2O\mathop \sum \limits_{i = 1}^{n} d\left( {p_{i} } \right)} \right) + O\left( {nO - 2\mathop \sum \limits_{i = 1}^{n} r\left( {p_{i} } \right)} \right)} \right]}$$

Algorithm I, referred to as RMSE, represents a key step in the feature evaluation process within the proposed framework. This algorithm calculates RMSE for a given feature.

**Algorithm I:**
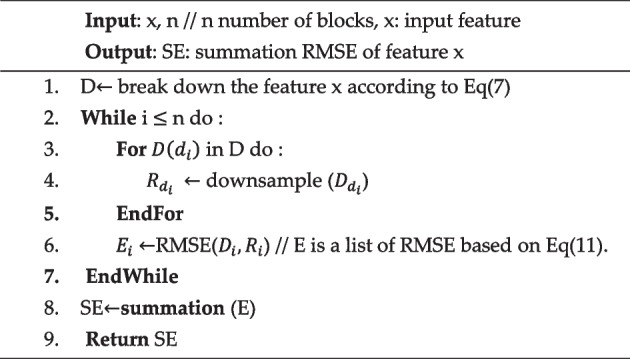
RMSE

The algorithm starts with inputs such as the feature vector ($x$) and the number of blocks n and aims to calculate the cumulative RMSE for each block of the feature. The algorithm is iterative and starts by dividing the feature x into blocks represented by (D). In each iteration, a down-sampling operation is performed for each block $D({d}_{i})$ in the set of blocks (D), resulting in the derivation of down-sampling representations, denoted ${{R}_{d}}_{i}$. Then, the RMSE for each block (${D}_{i}$) is calculated and stored in a list (E), which evaluates the accuracy of the sampled representation of the original data. Upon completing the loop, the algorithm culminates with summating all RMSE values stored in list (E). The cumulative result is assigned to the variable SE, encapsulating the overall RMSE for the feature.

In essence, Algorithm I: RMSE captures a crucial step in feature quality assessment by evaluating the accuracy of down-sampling representations using the RMSE metric. This metric serves as an indispensable criterion for selecting features with optimal performance characteristics and contributes to the improved predictive ability of the proposed system.

### Feature selection approach in FFS

The feature selection in the proposed FFS is demonstrated in process 4, as shown in Fig. 3. Algorithm II illustrates the procedure for selecting optimal based on low RMSE. It takes the $SE$ value and considers a predetermined percentage ($p$) for feature selection. The proposed feature selection strategy sorts features in ascending order according to $SE$ and selects the top $p$ as optimal features.

**Algorithm II:**
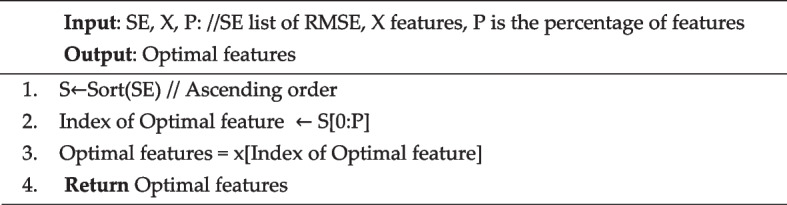
Feature selection

### Prepare train-test and evaluate features

Preparing train testing and evaluating features step corresponding to processes 4 and 5 in Fig. 3. Process 4 selects the optimal features from train and test data based on the features indicated in Algorithm II. It prepares the fundamental data to train and evaluate machine learning. Process 5 tests and evaluates the pre-train machine learning algorithms through various evaluation metrics, including accuracy, precision, recall, and F1 score.

## Experiments and discussion

This section includes details of benchmark datasets, experimental results to investigate the impact of the proposed FFS on machine learning performance, and a comparison with a fullset (without feature selection). Finally, the proposed model is compared with some feature selection studies.

### Dataset

Table 2 provides a comprehensive overview of the dataset details employed in the testing and evaluating of the proposed fractal feature selection (FFS) methodology. The complexity of data depends on the number of features and classes increases; an inverse relationship exists with the number of instances, leading to heightened data complexity (O). Equation (12) calculates the complexity of the dataset [2].

13 $$O = \frac{C.F}{I}$$

where C is the number of classes, F is the number of features, and I is the count of instances.

**Table 2:** Dataset details and complexity metrics [37]

Dataset name	Data details					O
	field	Type	Instance	Features	Class	
ALLAML	Biological	Discrete	72	7129	2	99.014
COLON	Biological	Discrete	62	2000	2	64.516
Lung_discrete	Biological	Discrete	73	325	7	31.164
Lung	Biological	Continuous	203	3312	5	81.576
Lymphoma	Biological	Discrete	72	7070	2	196.389
TOX_171	Biological	Continuous	171	5748	4	134.456
WarpPIE10P	Image	Continuous	210	2420	10	115.238
Orlraws10P	Image	Continuous	100	10,304	10	1030.4
CLL_SUB_111	Biological	Continuous	111	11,340	3	306.487
GLI_85	Biological	Continuous	85	22,283	2	524.306

### Evaluation metrics

This section centers on key evaluation metrics integral to machine learning and data science: Accuracy, precision, recall, and F1 score.

1. *Accuracy* quantifies the proportion of accurately predicted observations relative to the total observations, reflecting the model's predictive capacity. With true positives (TP), true negatives (TN), false positives (FP), and false negatives (FN) in focus, Eq. (14) computes accuracy using the formula:
  14 $$Acc = \frac{TP + TN}{{TP + TN + FP + FN}}$$
2. *Precision* pertains to the ratio of TP instances to all positive outcomes, encompassing incorrect identifications. Particularly valuable in cases where the ramifications of false positives hold significance, as seen in medical diagnoses, Eq. (15) computes precision.
  15 $${\text{Pre }} = \frac{TP}{{TP + FP}}$$
3. *Recall*, also labelled sensitivity, signifies the ratio of TP instances to the total count of samples that should have been classified as positive. It gauges a model's ability to detect all positive occurrences. Equation (16) calculates recall.
  16 $${\text{Rec}} = \frac{{{\text{TP}}}}{{{\text{TP}} + {\text{FN}}}}$$
4. *F1-score* emerges as a pivotal measure, encapsulating the harmonic mean of precision and recall. This metric balances the two, proving especially beneficial for imbalanced datasets. Equation (17) computes the F1-Score.
  17 $${\text{F1 - S}} = 2*\frac{{\text{Pre * Rec }}}{{\text{Pre + Rec}}}$$
5. *Correlation coefficient (*$r$*):* The correlation coefficient ($r$) quantifies the strength and direction of the linear relationship between two variables. It ranges from  − 1 (perfect negative correlation) to (perfect positive correlation), with 0 indicating no linear correlation. The correlation coefficient measures the strength of the linear relationship between two variables. A high correlation coefficient indicates that the two variables are strongly related, while a low correlation coefficient indicates that the relationship is weak. Equation (18) computes the correlation coefficient.
  18 $${\varvec{r}} = \frac{{\sum \left( {x_{i} - \overline{x}} \right)\left( {y_{i} - \overline{y}} \right)}}{{\sqrt {\sum \left( {x_{i} - \overline{x}} \right)^{2} \sum \left( {y_{i} - \overline{y}} \right)^{2} } }}$$
  where ${x}_{i}$ represents the value of the $i$ th observation in the first variable, ${y}_{i}$ represents the value of the $i$ th observation in the second variable, $\overline{x}$ is the mean of the first variable's values and $\overline{y}$ is the mean of the second variable's values.

### Experimental results

To evaluate the effectiveness of any feature selection model, we must assess its efficiency and performance using comprehensive metrics like accuracy. Additionally, comparing the results with prior works in the feature selection era confirms the validity of the proposed model in overcoming challenges faced by previous models. Therefore, we divided the analysis into three subsections: Feature testing, system performance assessment using machine learning algorithms, and comparison with previous and current feature selection models.

#### Experimental evaluation and performance analysis

This section highlights the impact of the proposed FFS model on the performance of machine learning algorithms. The main parameters of the proposed FFS model include the number of blocks (n) and the proportion of figures selected from the model (p). These parameters are determined by experimenting with different values and selecting the optimal value. Table 3 shows the results of testing machine learning algorithms on the best optimal FFS parameters ($n,p$). The examined machine learning algorithms encompass Naive Bayes (NB), Decision Trees (DT), Random Forest (RF), and Support Vector Machine (SVM), all collectively referred to as (ML). Furthermore, it is noteworthy that the abbreviation ML corresponds to machine learning algorithms, whereas FFS' signifies the amalgamation of the machine learning algorithm with the proposed feature selection model.

**Table 3:** Comparison of machine MLs with and without the proposed FFS model based on accuracy

Dataset	FFS parameters						Accuracy (%)					
	n	p	NB		DT		RF		SVM		KNN	
			ML	FFS’	ML	FFS’	ML	FFS’	ML	FFS’	ML	FFS’
ALLAML	2	16	89.03	91.25	86.67	91.25	90.48	100	73.33	89.03	89.03	89.03
COLON	10	21	43.77	52.15	69.23	80.18	84.21	94.74	61.54	73.2	74.54	94.74
Lung_discrete	4	81	66.68	75.84	66.68	77.24	76.19	95.24	66.68	66.68	86.67	95.24
Lung	2	36	75.37	80.74	75.61	88.04	81.82	90.91	70.73	72.68	72.68	90.91
Lymphoma	10	16	45.39	55.1	60.18	70	70.28	90.00	55.82	67.28	65.00	80
TOX_171	2	86	74.29	86.25	57.14	66.43	75	96.15	34.29	44.89	74.29	96.15
WarpPIE10P	10	6	90.24	93.65	83.33	83.22	76.19	93.65	52.86	63.65	88.62	93.65
Orlraws10P	10	21	80.99	91.45	61.44	77.14	79.31	100	50	63.05	91.45	91.45
CLL_SUB_111	2	26	69.56	86.97	65.22	76.32	69.57	86.97	34.78	45.22	65.22	86.97
GLI_85	4	36	70.59	71.28	76.47	96.55	79.31	96.55	64.71	78.24	88.24	88.24

The proposed Feature Selection (FFS) technique, guided by optimal (n,p) criteria, has identified and selected the most relevant features from each dataset. Specifically, the selected feature counts are ALLAML-1141, COLON-420, lung_discrete-263, lung-1192, lymphoma-1131, TOX_171-4943, warpPIE10P-145, orlraws10P-2164, CLL_SUB_111-2948, and GLI_85-8022. The SVM algorithm showed lower optimization results when applied as an objective function of the proposed traits test model because it could not cope with high dimensions, even though the proposed model reduced the dimensions by a significant percentage. However, in some cases, the algorithm did not achieve a significant improvement, such as at the beginning of TOX _171, where the percentage improvement was negligible (from 34.29 to 44.89), and also in the case of Lung_discrete, where the SVM saw no apparent progress. It is worth noting that in the first case, SVM accuracy was 86%, whereas in the second case, it was 81%. These results reflect the challenges of using the SVM algorithm in high-dimensional environments and show that some settings require more appropriate algorithms for large dimensions to achieve better performance.

**Fig. 4:**
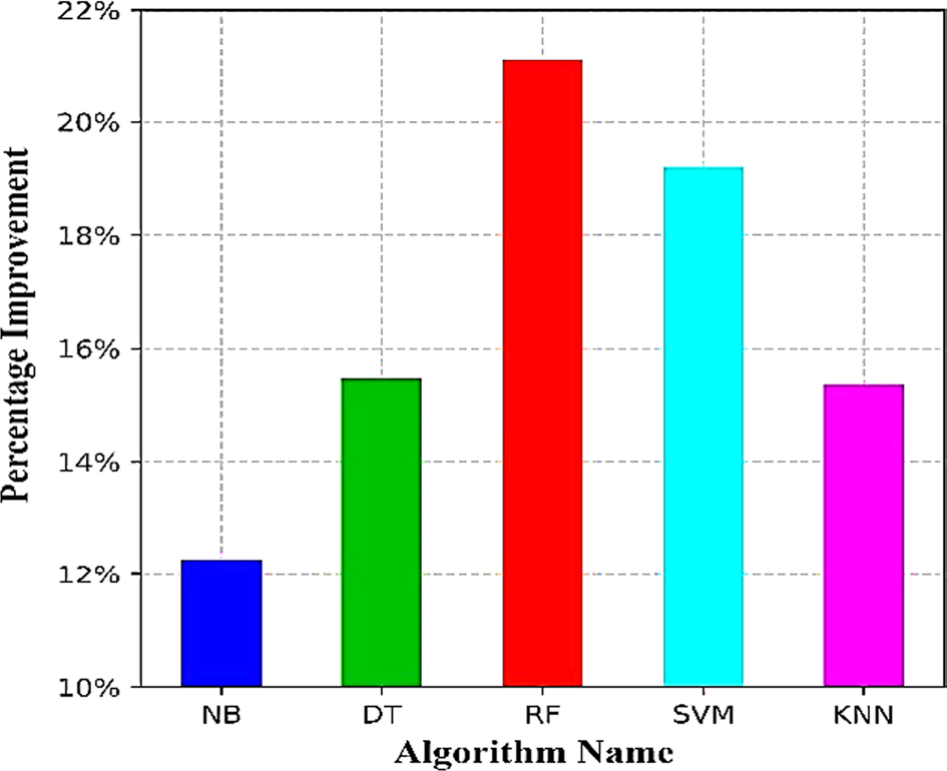
Comparative average percentage improvement of NB, DT, RF, SVM, and KNN

In the proposed model, the ML algorithm that achieves the highest accuracy is used as a predictive tool with the proposed FFS model. Table 3, the RF algorithm significantly improved performance on most test data. Improved ratios were observed for RF, enhancing the ML algorithm when using the proposed FFS. Ratios were 12.27%, 15.47%, 21.12%, 19.20%, and 15.36% for NB, DT, RF, SVM, and KNN algorithms. The proposed model development approach highlights the significant performance improvement of four AI learning algorithms (RF, KNN, NB, and DT) on specific attributes, as shown in Fig. 4.

Table 3 and Fig. 4 demonstrate that the RF algorithm's performance has significantly improved, achieving the highest accuracy and an enhanced level of pristine data. Consequently, a detailed analysis of its behaviour using evaluation metrics becomes essential. In this regard, Table 4 presents the experimental outcomes of the RF algorithm on the complete dataset and feature selection through FFS.

**Table 4:** Comparison of RF with and without the proposed FFS model

Dataset	FFS parameters	Accuracy								
	n	p (%)	Fullset				FFS			
			Acc	Prc	Rec	F1-s	Acc	Prc	Rec	F1-s
ALLAML	2	16	90.48	92.86	90.48	90.65	100	100	100	100
COLON	10	21	84.21	84.51	84.21	84.36	94.74	95.26	94.74	95
Lung_discrete	4	81	76.19	66.02	76.19	70.74	95.24	96.43	95.24	95.83
Lung	2	36	81.82	67.68	81.82	74.08	90.91	92.05	90.91	91.47
Lymphoma	10	16	70	57	70	60.75	90	86.81	90	78.16
TOX_171	2	86	75	84.03	75	79.26	96.15	96.44	96.15	96.30
WarpPIE10P	10	6	76.19	91.70	76.19	83.23	93.65	96.01	93.65	94.82
Orlraws10P	10	21	79.31	88.97	79.31	83.86	100	100	100	100
CLL_SUB_111	2	26	69.57	69.57	69.57	69.57	86.97	88.30	86.96	86.72
GLI_85	4	36	79.31	85.52	79.31	82.30	96.55	97.41	96.55	96.98

Table 4 presents a detailed assessment of the RF algorithm's performance in feature selection. Accuracy values demonstrate the algorithm's proficiency in classification tasks, with improvements achieved through the proposed feature selection strategy (FFS). FFS enhances precision, recall, and F1-score values across various datasets. The proposed model effectively selects correlated traits related to data objectives, characterized by high correlation with the targeted class. The close correlation between proposed FFS outcomes enhance classification and prediction accuracy, especially in the case of RF.

**Fig. 5:**
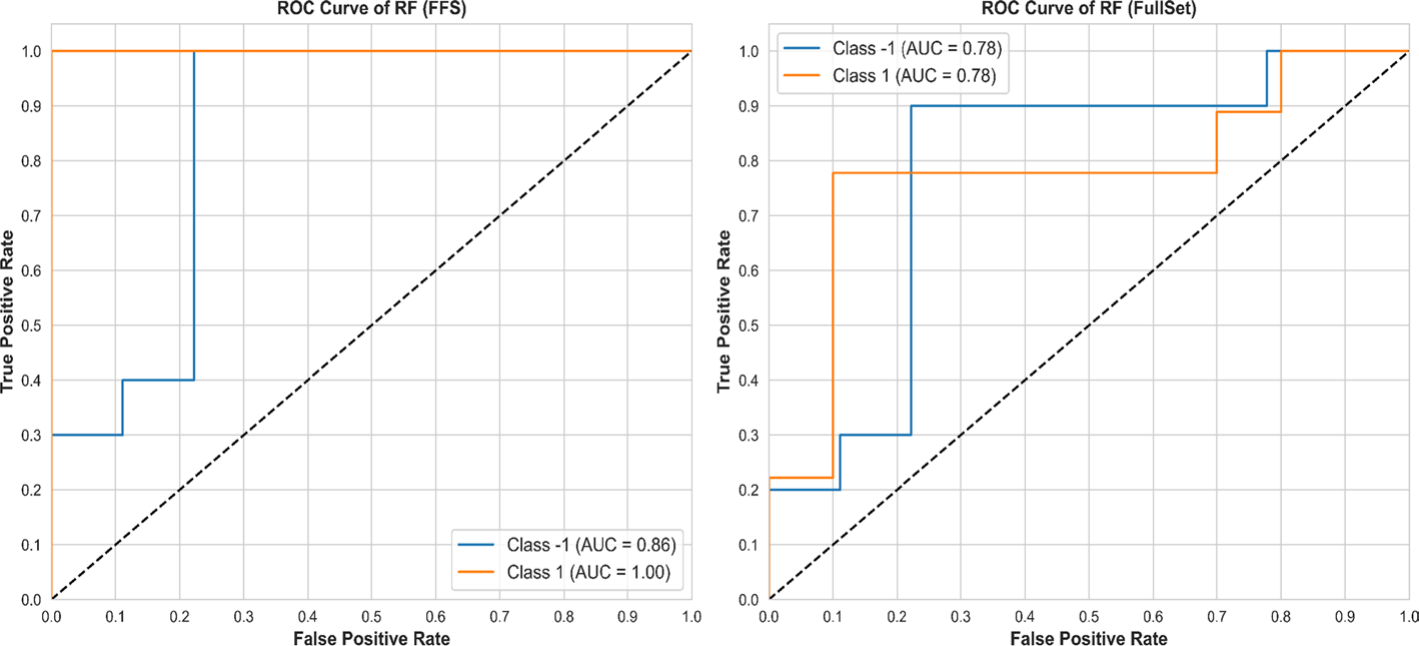
ROC Results of **COLON**

**Fig. 6:**
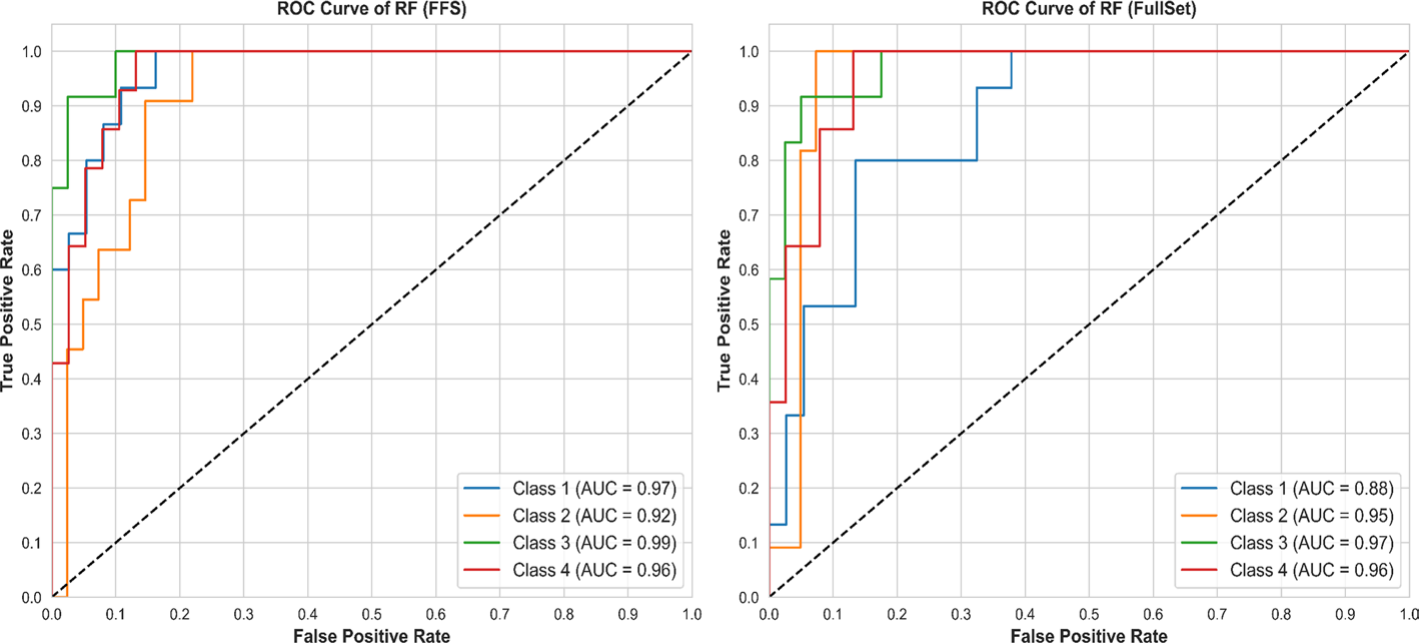
ROC results of **TOX_171**

Adding Receiver Operating Characteristic (ROC) analysis to evaluate RF algorithm performance offers insights into classification capabilities across different thresholds. Figures 5 and 6 illustrates the outcomes of ROC analysis conducted on a selection of experimental trials using the tested dataset.

Clearly, the proposed FFS have significantly improved the ability of RF to classify and discriminate between different datasets, even with varying complexity and imbalance of classes. The receiver operating characteristic curves (ROC) show an overall improved performance in various experimental trials, highlighting the robustness and adaptability of the algorithms. These results highlight the practical utility and effectiveness of the algorithms in optimizing classification results and contribute to more accurate and reliable prediction models in various scenarios.

#### Experimental features selection validity

This section compares the validity of the features of the proposed FFS model and the fullset according to the correlation coefficient. It calculates in three strategies: First, it shows the correlation coefficients between different characteristics (F-F), which provide information about their potential interdependencies or unique contributions; Second, the table shows the correlation coefficients between these features and the target label (F-L), illustrating their relevance for prediction; Finally, the average absolute correlation coefficient between the features and features and the target label is presented (O-F). Table 3 compares the correlation coefficients between standard features and the features selected by the FFS model.

From Table 5, it is clear that the proposed FFS improve the correlation coefficient of output features. This improvement reflects the efficiency of the developed system in selecting features that relate to the target of the data. The increase in the correlation coefficients among features (F-F) and features-label (F-L) shows the increasing ability of the model to capture the high correlation features.

**Table 5:** Comparison of Correlation Coefficients between Standard Features and FFS-Selected Features

C	Fullset			FFS		
	F-F	F-L	O-F	F-F	F-L	O-F
ALLAML	0.399	− 0.163	0.281	0.681	− 0.598	0.6395
COLON	0.382	− 0.145	0.263	0.576	− 0.47	0.523
Lung_discrete	0.122	0.184	0.153	0.556	0.419	0.4875
Lung	0.36	0.167	0.263	0.494	0.324	0.409
Lymphoma	0.281	− 0.118	0.2	0.402	0.317	0.3595
TOX_171	0.119	0.143	0.131	0.612	0.547	0.5795
WarpPIE10P	0.236	− 0.148	0.192	0.454	− 0.273	0.3635
Orlraws10P	0.185	0.02	0.103	0.685	0.486	0.5855
CLL_SUB_111	0.381	0.259	0.32	0.412	0.383	0.3975
GLI_85	0.401	0.182	0.292	0.684	0.484	0.584

**Fig. 7:**
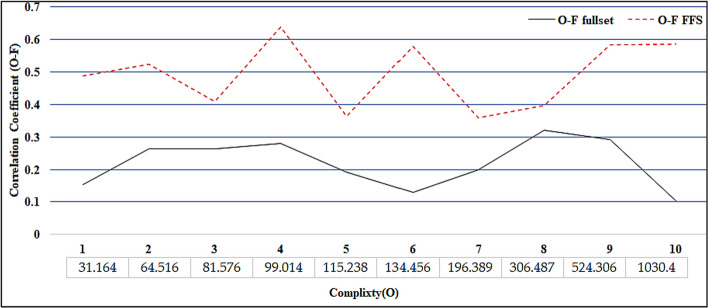
Comper the correlation coefficient (O-F) between fullSet and features selection by FFS

Figure 7 compares the average correlation coefficient between the fullset and the features selected by the proposed FFS.

After examining Fig. 7, it is evident that the FFS strategy selects highly correlated features, can handle complicated data structures, and extracts reliable features. The plotted data prove that the FFS consistently improves correlation values across various datasets.

### Comper with other studies

This section provides a comprehensive overview of the current research landscape in feature selection. Table 6 compares the accuracy of the proposed FFS model with other studies on different datasets. The bold text in Table 6 indicates the highest value within each column, which represents the best result.

**Table 6:** Compare the proposed FFS model with other studies in term of accuracy

Refs.	Publishing year	ALLAML	COLON	Lung	lymphoma	TOX_171	warpPIE10P	orlraws10P	CLL_SUB_111	GLI_85
Al-Shammary et al. [2]	2021	x	87.00	x	**100**	x	x	x	x	x
Wei et al. [12]	2020	98.86	x	x	95.34	80.69	**98.33**	86.46	85.95	95.02
Gao et al. [28]	2018	x	83.57	x	x	63.23	86.67	x	x	x
Gao et al. [29]	2020	94.08	78.33	x	74.66	x	67.32	x	x	83.02
Zhang et al. [30]	2021	x	92.41	90.12	93.79	x	x	x	70.92	x
Wang et al. [31]	2023	97.17	x	**91.42**	x	93.39	94.62	95.10	**96.13**	90.76
Proposed FFS		**100**	**94.74**	90.91	**90**	**96.15**	**93.65**	**100**	**86.97**	**96.55**

The proposed FFS model outperformed the model in [2] on one dataset due to its structured and systematic approach, which ensures higher reliability and credibility of the results. In contrast, the model in [2] relies on a randomization approach, which may lead to unstable results, potentially undermining its reliability. Comparing the performance of the proposed model with the model in [12], it outperformed the common dataset by 88%. The proposed FFS model outperformed the models in references [28–30] and [31] by 78% on shared datasets with the proposed FFS. Table 6 shows that the proposed FFS outperforms the comparative studies.

To sum up, the thorough analysis and discussion of the results show that the proposed FFS model is compelling performance and highly accurate. The results demonstrate that FFS significantly improves the accuracy of machine learning algorithms (KNN, RF, NB, and DT) on diverse and complex datasets. Moreover, the FFS method has a clear advantage in selecting and testing features with high correlation with data objects, which makes it useful for real-world applications.

## Discussion

High-dimensional problems present a significant challenge in machine learning. As dimensionality increases, it becomes increasingly difficult to distinguish between data categories, leading to issues with model interpretability and overfitting. High-dimensional data is inherently intricate due to several factors:

1. *Presence of irrelevant information or “Noise”*: High-dimensional datasets often encompass extraneous or non-pertinent information, termed as "noise". Such unrelated data can mislead models, resulting in inaccurate outcomes.
2. *Complex inter-feature relationships*: Even among features that may be interrelated, the relationships can be nuanced and multifaceted. Identifying and analyzing these intricate relationships heighten the challenges of understanding and interpreting the data.

In fractals, a central tenet posits that patterns recur at differing scales. This principle suggests that when one examines a minuscule segment of a fractal and juxtaposes it with a more significant portion of the same fractal, the patterns observed will bear striking resemblance. According to Eq. (5), the R and D describe relationships between data across different scales that can be discerned. The proposed fractal feature selection (FFS) model offers a novel approach to this issue. It partitions features into blocks, measures similarity using the Root Mean Square Error (RMSE), and determines feature importance based on low RMSE values. This approach reduces the randomness and unpredictability inherent in traditional models and uncovers hidden relationships and nuances within the data.

Integrating the proposed Feature Selection method (FFS), the Random Forest (RF) algorithm demonstrates enhanced performance over other algorithms, including SVM, NB, and KNN. By employing ensemble learning, RF notably increases the stability and accuracy of predictions. Moreover, its adeptness at managing extensive datasets provides a significant advantage. While versatile in handling quantitative and categorical variables, the RF algorithm doesn't lean on specific assumptions. Its capacity to efficiently manage a range of data types, coupled with the interpretative advantages of FFS, solidifies its prominence in numerous scientific contexts.

## Conclusion

Bioinformatics combines biological data with analysis techniques for scientific research, including biomedicine. It depends on the analysis of the gene activity in the cell. Gene expression profiling (GEP) is a powerful tool that generates thousands of features, but not all are relevant to a particular cancer. Therefore, machine learning needs feature selection to improve cancer detection and classification. Proper feature selection is critical when working with big data and high dimensions to avoid overfitting and data noise and ensure AI's effectiveness. The correlation coefficient calculates the degree of relationship between different features and the information carried by those features, which helps improve the classification accuracy of machine learning algorithms. The higher the correlation coefficient, the higher the correlation between the attribute and the target of the data. When the model breaks down a feature into multiple blocks, each block is associated with one of the available data categories. The greater the similarity between these blocks, the more closely they are associated with a particular data target, provided the similarity values are not zero. Therefore, this work proposes using fractal concepts to optimize the features of the high-dimensional problems. The proposed fractal feature selection (FFS) model divides features into blocks, measures the similarity between blocks using Root Mean Square Error (RMSE), and determines the importance of features based on low RMSE. It's important to note that a limitation of the FFS model is that its performance may decrease as the number of classes decreases, which is associated with an increase in RMSE values. To improve the proposed FFS model in future work, A primary direction we anticipate is the integration of FFS with advanced computational techniques. As data complexities grow, amalgamating FFS with state-of-the-art machine learning, such as deep learning architectures, can potentially amplify feature selection capabilities for neural networks. This amalgamation can be particularly advantageous for handling the increasing size of datasets, focusing on enhancing the scalability of FFS. It will be imperative to explore how FFS performs when faced with vast data realms and discern the modifications necessary to cater to them efficiently.

## Data Availability

The data supporting this study's findings are openly available in the reference [34].
